# Analysis of Stiffness and Energy Consumption of Nonlinear Elastic Joint Legged Robot

**DOI:** 10.1155/2020/8894399

**Published:** 2020-07-11

**Authors:** Dongliang Chen, Jindong Zhang, Xutao Weng, Yunjian Zhang, Zhonghui Shi

**Affiliations:** College of Mechanical and Electrical Engineering, Harbin Engineering University, Harbin 150001, China

## Abstract

In order to reduce the energy consumption of the legged robot in walking, this paper designs a kind of nonlinear elastic joint from the flexible variable-stiffness joint based on the mammal walking on the limb and optimizes the leg structure of the legged robot. The motor is rigidly connected to the articulated lever. When the lever is accelerated or decelerated, the elastic unit is introduced. The system can be considered as a special variable-rate elastic system. This paper will study it from theory and simulation experiments. Based on the dynamic analysis, a functional relationship between the output torque and the torsion spring stiffness and between the energy consumption and the torsion spring stiffness was established. By finding the extremum, the two optimum torsional spring stiffness that can minimize the required output average torque and the energy consumed during one cycle of motion were deduced. The results show that using this design in a reasonable position can effectively reduce the energy consumption of the system and can achieve up to a 50% reduction in energy consumption.

## 1. Introduction

Compared with the wheeled robot, the foot robot has great advantages in adaptability to the external complex environment and has great research value [1]. With the increase of the number of feet, the structure of the foot robot is more redundant, but it improves the fault-tolerant ability of the robot: the damage of one leg will not have a great impact on the robot, it can move smoothly with the cooperation of other legs. However, the foot robot needs to coordinate its limbs, and its motion efficiency is relatively low, and its energy loss is relatively large. In the process of movement, the friction loss between the body and the ground as well as between the mechanisms will also be dissipated by the heat generated by the driving winding due to the need to maintain a fixed joint angle in the process of standing still. Therefore, for the foot robot, how to reduce energy consumption to improve energy utilization is particularly important, which will bring great performance improvement to the foot robot.

In recent years, the research on energy-saving optimization of foot robot can be classified into the following four categories: (1) study the energy conversion of bionic joint by analyzing the physiological structure of leg joint of foot animal [2]; (2) optimize the end trajectory curve under different conditions [3]; (3) reasonable gait planning and optimal gait generation parameters [4]; and (4) how to reasonably distribute the foot force [5]. A large number of experiments have shown that human beings and some creatures use their own physiological structure to complete energy transfer and transformation when they are in motion, which is a highly coupled function brought by evolution and the body [6]. For example, in the movement system composed of human bones, muscles, and tendons, the flexible components of muscles and tendons produce the effect similar to that of springs, which are regularly shaped in motion. Variable can reduce energy consumption and overall impact of the system, which can be approximately considered as a load spring inverted pendulum slip model [7, 8]. Inspired by this idea, this paper introduces the spring design idea to the joint motion of the foot robot for energy conversion. Nowadays, many scholars have studied the elastic joint of robot and discussed the structure and control method of the elastic joint, but most of them focus on the performance research of damping and reducing the impact force [9]; this paper will study the energy consumption brought by it.

However, the flexibility of the robot joint has a significant impact on the positioning accuracy of the legs and the accuracy of the end of the trajectory when the robot moves [10], and the degree of freedom of its control system will increase by one level, resulting in its control more complex and easy to lose stability. Therefore, a large number of scholars have studied the impact on the control model after introducing the flexibility of the joint. Nowadays, there are many significant achievements in system modeling [11] and dynamic modeling [12] of robot with elastic joints. However, for the foot robot with high redundancy, such as the six-legged robot, the lack of accuracy of the foot end trajectory of one leg is not enough to cause the stability imbalance of the whole robot, but the resulting energy consumption is reduced, which can greatly improve the endurance of the foot robot. Secondly, most of the current research uses linear elastic elements and will participate in the whole movement. In this paper, a joint motor is used to drive the joint directly, and a torsion spring is installed at the joint which is in contact with the bar. By improving some elastic joints, a nonlinear elastic system is formed. Only when the joint motor decelerates to the stop and the initial speed is zero to the acceleration, the elastic joint will start to play an obvious role. In this way, it can help the joint motor decelerate in the deceleration process and accelerate in the acceleration process, so as to achieve an energy conversion function and reduce energy loss. In the following chapters, we will start with a simplified model of nonlinear elastic connections. Through dynamic analysis, the relationship between motor output torque and spring stiffness coefficient is found. Then, through the analysis of energy consumption, the value of joint stiffness coefficient is found. After that, we will design some simulation experiments to verify the effect of nonlinear elastic connection on the system. Finally, the relevant conclusions are summarized.

## 2. Dynamic Modeling and Analysis

**Figure 1:**
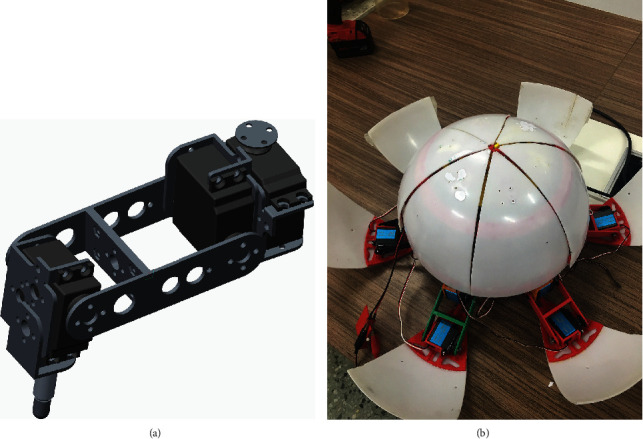
(a) Legged robot single-leg model; (b) a hexapod robot prototype.

The single-leg structure of the legged robot model and the prototype are shown in Figure 1. The robot can switch arbitrarily between a hexapod robot and a spherical robot. Each leg has three degrees of freedom and three joint motors. This paper focuses on the second joint, that is to say, only the leg joints are subjected to a simulation experiment with nonlinear elastic joints. For each revolute joint, a similar dynamic model will be generated when the torsion spring of the elastic joint is assembled in a suitable position. They have the same properties and only differ in numerical values.

**Figure 2:**
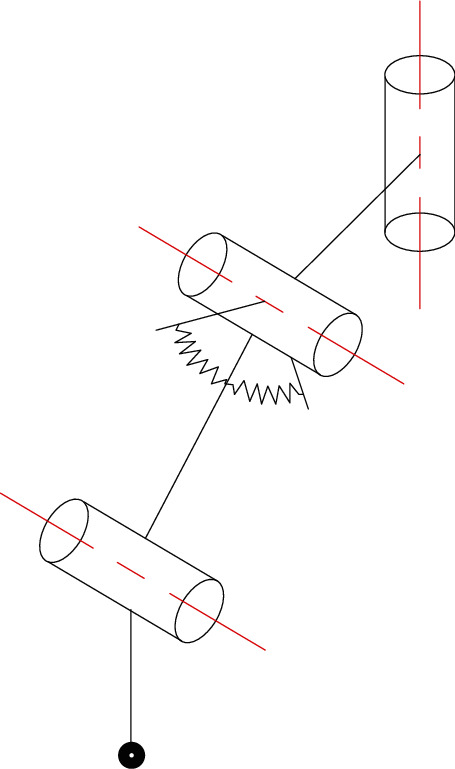
Single-leg simplified model with nonlinear elastic joints.

The single-leg simplified model with nonlinear elastic joints is shown in Figure 2. Since this paper only does theoretical analysis and simulation, in order to simplify the model, the ideal position is taken during assembly without actual assembly. Some elastic joints are horizontal and symmetrical with the axis of the revolute joint.

Through dynamic modeling, the relationship between joint torque and joint motion parameters of a nonlinear elastic joint robot can be deduced. At the same time, the relationship between energy consumption and joint movement can also be deduced. This paper uses the Lagrangian method to derive the dynamic equations of the elastic joint model. The elastic part of the torsion spring has no moment of inertia and is elastically deformed only in one direction, ignoring the damping of the elastic unit.

**Figure 3:**
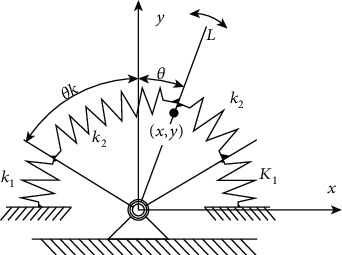
Simplified nonlinear elastic joint mechanism.

Assume that the initial momentum of the system is zero. In the theoretical derivation and simulation experiments, this paper concludes that the trajectory is accurately tracked, and there is no error such as offset and phase difference.  Figure 3 shows the simplified nonlinear elastic joint mechanism. The parameters of the mechanism diagram are shown in Table 1; the torsion spring that mainly acts in the elastic joint is defined as the main torsion spring k_1_, and its stiffness is far greater than that of the secondary torsion spring k_2_ that only reduces the impact, that is, k_1_ is much larger than k_2_ and k_2_ ≈ 0, and the barycentric coordinate of the lever is set as (x, y).

The joint space is divided into two parts: an elastic joint with a very small stiffness and an elastic joint with a large stiffness. In order to simplify the equation, it is necessary to make the following assumption. In the collision process, the collision is stable and the time is not counted. The collision is surface contact. The position and direction of the lever and the contact surface do not change during the collision. The inertia of each component does not change, and the deformation does not occur during the collision. Lagrangian equation is still established during collision [13].

As shown in Figure 3, we separately model the dynamics of different stiffness. In an ideal state, assuming that the rigid joint driving torque is M(*θ*), the torque of the torsion spring and the joint rotation angle in the elastic system are, respectively, the functions of the relationship T_k1_(*θ*) and T_k2_(*θ*). When the lever has not yet reached the torsion spring with the large stiffness, the required torque for the joint can be given by

(1) $$\begin{array}{c} T_{q1}\left(\theta \right)=M\left(\theta \right)+T_{k2}\left(\theta \right). \end{array}$$

The required torque after the lever comes into contact with the large stiffness torsion spring can be given by

(2) $$\begin{array}{c} T_{q2}\left(\theta \right)=M\left(\theta \right)+T_{k1}\left(\theta \right)+T_{k2}\left(\theta \right). \end{array}$$

After the trajectory curve is determined, the joint output torque curve in each period is also determined, that is, the relationship between T_q1_, T_q2_ and time is a determined curve. The sum of the output torque of the joint and the torque generated by the elastic joint is a certain value at a certain point of time. At this time, the greater the torque generated by the elastic joint, the smaller the required output torque of the joint.

However, in order to make the dynamic model more accurate, the two parts of the joint space are now integrated into a dynamic analysis. The D-H method [14] is used to establish the coordinate system of the joint. Only the second joint is taken as the research object.

The kinetic equation of the robot is represented as follows:

(3) $$\begin{array}{c} E=\frac{1}{2}m\left({\dot{x}}^{2}+{\dot{y}}^{2}\right)+\frac{1}{2}l{\dot{\theta }}^{2}, \end{array}$$

where x, y can be given by

(4) $$\begin{array}{c} x=l\sin\theta ,\\ y=l\cos\theta . \end{array}$$

Equation (4) can be rewritten as

(5) $$\begin{array}{c} E=\frac{1}{2}ml^{2}{\dot{\theta }}^{2}+\frac{1}{2}I{\dot{\theta }}^{2}. \end{array}$$

Gravity energy can be given by

(6) $$\begin{array}{c} P_{1}=mgy=-mg\text{l}\cos\theta . \end{array}$$

There is almost no elastic potential energy in the system before the torsion spring with a large stiffness is touched, and the stiffness coefficient can be regarded as linear after the main torsion spring is contacted. So it can be considered as a special nonlinear variable stiffness elastic model in one cycle. The elastic potential energy can be given by

(7) $$\begin{array}{c} P_{2}=\frac{1}{2}k\left(\theta \right){\left(\alpha \Delta \theta \right)}^{2}, \end{array}$$

where *α* is the angle conversion factor when the torsion spring and the rotation center do not coincide with each other, and *α* keeps the value of the rotation angle of the torsion spring and the rotation angle of the joint united. Δ*θ* is the rotation angle of the torsion spring with respect to the joint and can be approximated as the joint rotation angle *θ*, because the lever and the motor are rigidly connected. k(*θ*) is a function of the nonlinear torsion spring for the joint rotation, which can be expressed in this paper by a piecewise function:

(8) $$\begin{array}{c} k=\left\{\begin{array}{cc} 0, & \left(\left|\theta \right|-\theta_{\text{k}}\leq 0\right),\\ k_{1}, & \left(\left|\theta \right|-\theta_{\text{k}}>0\right). \end{array}\right. \end{array}$$

The total potential energy can be given by

(9) $$\begin{array}{c} P=P_{1}+P_{2}=-mgl\cos\theta +\frac{1}{2}k\left(\theta \right){\left(\alpha \Delta \theta \right)}^{2}. \end{array}$$

The Lagrangian operator can be given by

(10) $$\begin{array}{c} L=E-P=\frac{1}{2}{ml}^{2}{\dot{\theta }}^{2}+\frac{1}{2}I{\dot{\theta }}^{2}+mgl\cos\theta -\frac{1}{2}k\left(\theta \right){\left(\alpha \Delta \theta \right)}^{2}. \end{array}$$

The Lagrangian kinetic equation can be given by

(11) $$\begin{array}{c} \frac{\partial L}{\partial \theta }=-mgl\sin\theta \pm \dot{k}\left(\theta \right)\left(\alpha \Delta \theta \right), \end{array}$$

(12) $$\begin{array}{c} \frac{d}{dt}\left(\frac{\partial L}{\partial \dot{\theta }}\right)={ml}^{2}\ddot{\theta }+I\ddot{\theta }. \end{array}$$

According to the Lagrange equation, the joint torque can be obtained as

(13) $$\begin{array}{c} M=\frac{d}{dt}\left(\frac{\partial L}{\partial \dot{\theta }}\right)-\frac{\partial L}{\partial \theta }={ml}^{2}\ddot{\theta }+I\ddot{\theta }+mgl\sin\theta \mp \dot{k}\left(\theta \right)\alpha \Delta \theta . \end{array}$$

Assume that the joint output rotation function is f(t), then the output torque after contact with the elastic joint can be given by

(14) $$\begin{array}{c} M_{k}={ml}^{2}\ddot{f}\left(t\right)+I\ddot{f}\left(t\right)+mgl\sin f\left(t\right)-k\left(f\left(t\right)\right)\alpha \left(f\left(t\right)-\theta_{k}\right). \end{array}$$

Equation (14) has the same conclusion as equations (1) and (2). Equation (14) shows that before the end of the stance phase and the swing phase, the robot needs a joint motor to decelerate. With the participation of the elastic joint, due to the compression of the torsion spring, the torsion spring will produce an increasingly larger countertorque. It can effectively reduce the output torque of the joint motor under the same combined torque. At the beginning of a new swing phase or stance phase, the joint motor output torque is required to provide sufficient acceleration to the joint. In the model with elastic joints, the torsion spring is compressed when the previous movement decelerates. So the torsion spring and the joint motor collectively output torque in the acceleration phase. When the required torque is the same, the output torque of the joint motor can be greatly reduced. Therefore, theoretically, the design of the elastic joint can reduce the output peak torque of the joint motor, which can effectively reduce the energy consumption of the robot during walking.

In order to obtain the best stiffness function of the torsion spring with the purpose of minimizing the output torque, differentiate equation (14) of time and let its value be zero:

(15) $$\begin{array}{c} \dot{f}\left(k\right)=\frac{{dM}_{k}}{dt}=0. \end{array}$$

k_M_ (*θ*) can be obtained from equation (15). If $\ddot{\text{f}}\left(k_{M}\left(\theta \right)\right)\geq 0$, then k_M_ (*θ*) (N∗m/rad) is the function of the torsion spring stiffness that minimizes the output torque under this input function.

For the robot's trajectory design, many scholars have done a lot of researches [15, 16], such as composite cycloids, modified elliptical trajectories, and classic multi-item fitting curves. For the joint tip trajectory planning with continuous smooth acceleration and the appropriate initial conditions for position and velocity, a simpler sinusoidal trajectory will be used, i.e.,

(16) $$\begin{array}{c} f\left(t\right)=A\sin\left(\omega \text{t}+\phi \right)+B. \end{array}$$

Equation (14) can be rewritten as

(17) $$\begin{array}{c} M_{k}=-\left({ml}^{2}+I\right)A\omega^{2}\sin\left(\omega t+\phi \right)+mgl\sin\left(A\sin\left(\omega t+\phi \right)+B\right)-k\left(A\sin\left(\omega t+\phi \right)+B\right)\alpha \left(A\sin\left(\omega t+\phi \right)+B-\theta_{k}\right). \end{array}$$

Differentiate equation (17) of time and ignore the effect of gravity. When *t* ∈ ((arcsin((*θ*_*k*_ − *B*)/*A*) − *ϕ*)/*ω*, *π*/2*ω*), equation (17) can be rewritten as

(18) $$\begin{array}{c} \dot{f}\left(k\right)=\frac{{dM}_{k}}{dt}=-\left({ml}^{2}+I\right)A\omega^{3}\cos\left(\omega t+\phi \right)-{Ak}_{2}\alpha \omega \cos\left(\omega t+\phi \right). \end{array}$$

Let equation (18) be equal to zero and get the result as follows:

(19) $$\begin{array}{c} k_{2}=\frac{\omega^{2}\left({ml}^{2}+I\right)}{\alpha }. \end{array}$$

If $\ddot{\text{f}}\left(k_{2}\right)>0$, then *k*_*M*_ = *k*_2_ is the optimal stiffness that will make the output torque the smallest torsion spring.

For different trajectories, the specific positions of the main torsion springs for joint contact need different analysis, and it is necessary to ensure that the torsion spring contacts the deceleration when the joint driving torque is greatly reduced, and a symmetrical acceleration torque is required. When using different driving torque curves for acceleration and deceleration, the situation will become more complicated and changeable.

## 3. Energy Consumption Analysis

Without considering other factors such as friction loss, the energy consumed by a single joint model with a nonlinear elastic joint in one exercise cycle can be expressed as

(20) $$\begin{array}{c} E=\int_{0}^{T}pdt=\int_{0}^{T}\tau \omega dt=\int_{0}^{T}M_{\text{k}}\dot{\theta }dt, \end{array}$$

where p is the joint drive power, *τ* is the joint drive torque, *ω* is the joint rotation angular velocity, and *θ* is the joint rotation angle. From equation (20), it can be seen that the energy consumption is related to the integral of the output torque and the angular velocity at this moment, and the output torque and the joint angular velocity are both functions of time. Equations (16) and (17) can be expressed as

(21) $$\begin{array}{c} E=\int_{0}^{T}\left(\left({ml}^{2}+I\right)\ddot{f}\left(t\right)\dot{f}\left(t\right)+\dot{f}\left(t\right)mgl\sin f\left(t\right)\right)-\dot{f}\left(t\right)k\left(f\left(t\right)\right)\alpha \left(f\left(t\right)-\theta_{k}\right)dt. \end{array}$$

If we want to obtain a stiffness k_E_ that minimizes E, then

(22) $$\begin{array}{c} g\left(k\left(f\left(t\right)\right)\right)=E. \end{array}$$

Let $\dot{\text{g}}\left(k\right)=0$ to get k_*E*_. If $\ddot{\text{g}}\left(k_{E}\right)>0$, then k_*E*_ is the demand.

Assume that the energy consumption ratio *ε* is an evaluation standard for energy consumption [17]. This assessment of energy consumption is based on the metabolic effects of the organism [18, 19]. The energy consumption ratio means that the unit of weight requires a unit of energy to dissipate the distance. The smaller the value is, the higher the energy consumption can be obtained.

(23) $$\begin{array}{c} \varepsilon =\frac{E}{m_{c}gS}, \end{array}$$

where m_c_ represents the total mass of the robot and S is the standard of the robot's moving distance.

The rotational movement of the joint is the main movement, so make some changes to equation (23) to conform to the simulation movement in this paper. Let the moving standard S = *θ* and the total weight be replaced with the moment of inertia about the rotation axis, equation (23) can be rewritten as

(24) $$\begin{array}{c} \varepsilon =\frac{\int_{0}^{T}M_{k}\dot{\theta }dt}{I\theta }. \end{array}$$

## 4. Simulation Experiments

### 4.1. Experiment Environment

**Figure 4:**
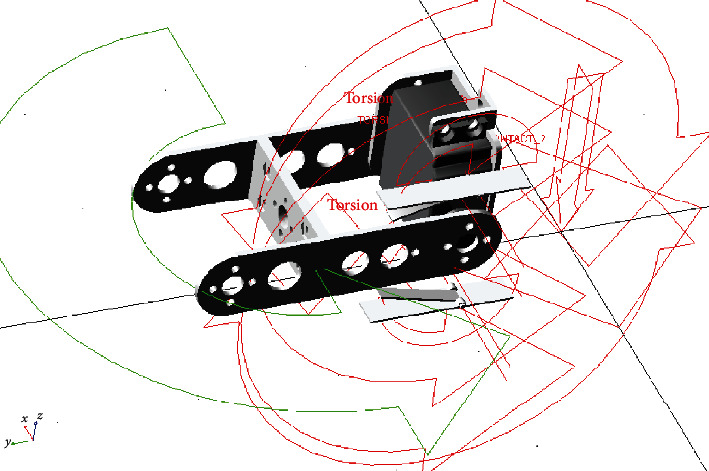
Simulation model of robotic leg with nonlinear elastic joint.

Using Adams software for simulation experiments, the experimental model was modeled in Creo software and imported into Adams, as shown in Figure 4. After removing the components that are not related to the movement, add the parameters of friction and force of the restraint and the motion parameter.  Table 2 is the setting of the frictional force parameter of the joint rotation; Table 3 lists the parameter setting of the contact force between the lever and the torsion spring; Table 4 is the setting of the secondary torsion spring physical parameters; Table 5 lists the parameters of the main torsion spring physical. The joint adopts a general point driving method. After inputting the endpoint trajectory, the required torque value of the joint is sampled. In order to keep the motor from no-load spinning, the end of the joint is loaded with an equivalent mass load. The trajectory function is shown in

(25) $$\begin{array}{c} f\left(t\right)=0.3\sin\left(2t\right), \end{array}$$

where t is time.

The model with a nonlinear elastic joint was set as the experimental group, and the model without the elastic joint was the control group. The physical dimensions and simulation parameters of the two models were basically the same, as shown in Table 6.

### 4.2. Simulation Experiment Results

**Figure 5:**
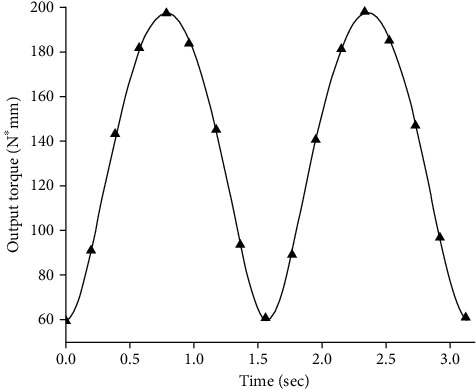
Required output torque of joint without nonlinear elastic joint.

**Figure 6:**
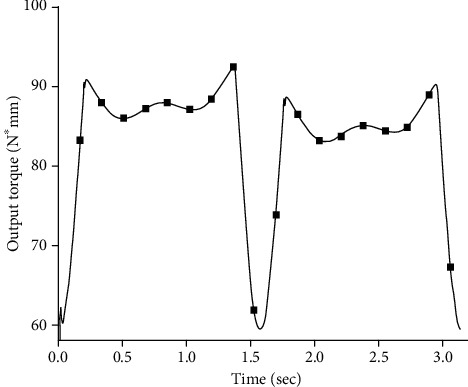
Output torque required at torque spring K = 400 N∗mm/deg.

The simulation experiments found that under the same quality and external conditions, the output torque required by the experimental group model compared to the control group model was greatly reduced. The simulation results of the required output torque at the joint of the control group model are shown in Figure 5, and the simulation results of the required output torque at the joint of the experimental group are shown in Figure 6.

**Figure 7:**
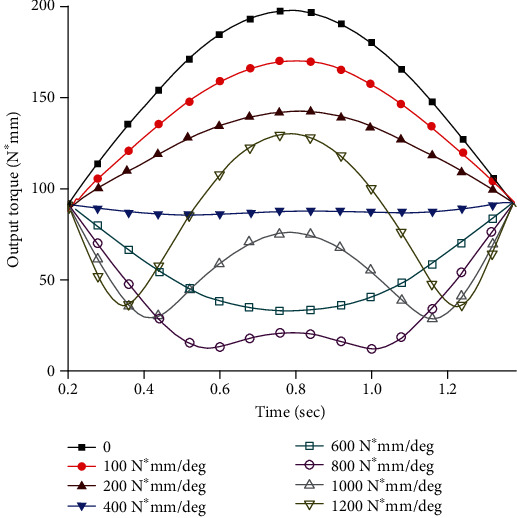
Required output torque for joints with different stiffness.

We changed the stiffness of the torsion spring and carried out the simulation experiment, as shown in Figure 7, which were the required output torque of the joint when the stiffness of the torsion spring was different.

It can be clearly seen from Figure 6 that due to the limitation of the function k(*θ*) in equation (13), the joint torque is a normal output before contacting the main torsion spring. In Figures 5 and 6, there is a coincident torque output value under different stiffness. Figure 7 show the torsion spring has a stiffness ranging from 100 N∗mm/deg to 1200 N∗mm/deg. The required output torque of the joint first slowly decreases to a certain value and then begins to rebound. This rebound phenomenon is most obvious when the joint gets close to the limit of rotation. The torsion spring in this case has an optimum stiffness that minimizes the required output torque. The trajectory parameters and the model physical parameters are brought into equation (19), where *θ*_k_ = 7.32 deg and *α* = 1.05, resulting in an optimum stiffness k_M_ = 713.547 N∗mm/deg.

**Figure 8:**
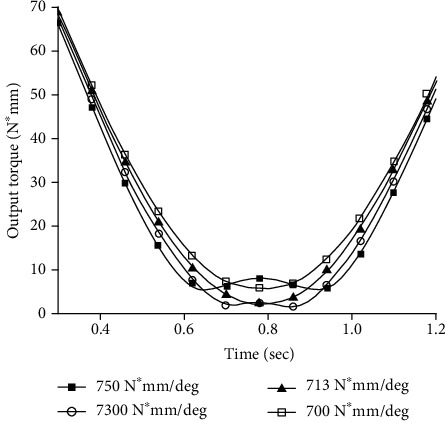
The required output torque of the joint around the theoretical stiffness k_M_.

The above k_M_ and nearby values were used as the stiffness of the simulation model. The results (k_M_) are shown in Figure 8. The experimental results showed that the simulation experiment was not exactly the same as the theoretical value and there was a certain error.

After several simulations around k = 713 N∗mm/deg, the simulation results showed that the required output torque was slightly compared with other stiffness at the same time around k = 740 N∗mm/deg. The following numerical calculation will verify its correctness.

**Figure 9:**
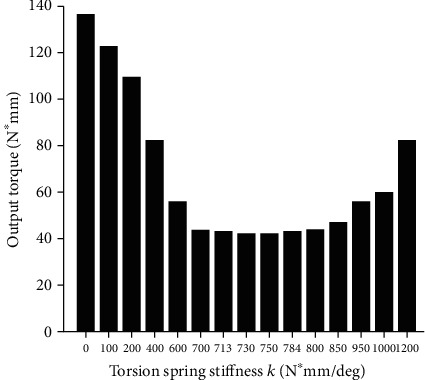
Average required output torque for different stiffness in one cycle.

**Figure 10:**
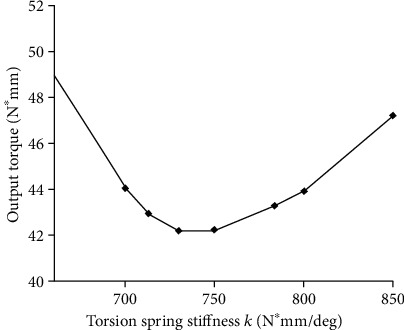
Average required output torque in one cycle around k_M_.

Matlab was used to process the discrete torque derived output torque M from the Adams. After using the trapez function to integrate its data over a period, and then dividing it by the total time in one cycle, the average output torque under different stiffness per unit time was obtained. As shown in Figure 9, the simulation result around the theoretical derivation k_M_ is shown in Figure 10.

Figure 10 shows that the average moment around k = 740 N∗mm/deg is the lowest, and the maximum error between the simulated value and the theoretical value is about 3.7%.

**Figure 11:**
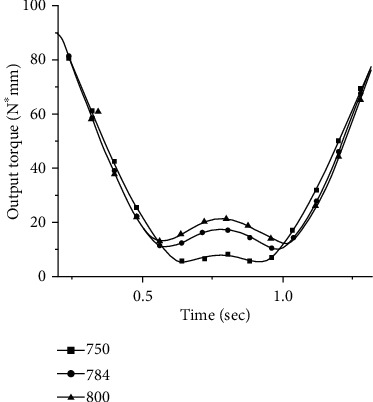
The required output torque of the joint under the theoretical stiffness around k_E_.

The trajectory curve was brought into equation (21). So that it was integrated over the interval *θ*_k_ to *π*/4, resulting in the torsional spring stiffness k_E_ = 784.717 N∗mm/deg that optimized the energy. The simulation results of the required joint output torque under the stiffness around k_E_ are shown in Figure 11.

**Figure 12:**
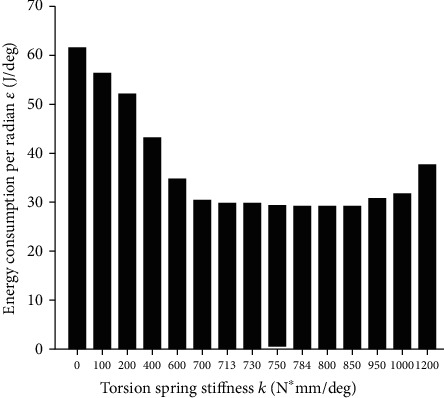
Energy consumption ratio under different stiffness.

**Figure 13:**
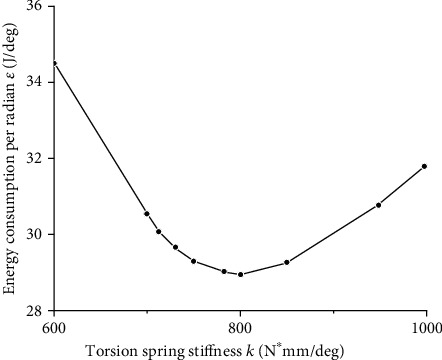
Energy consumption ratio under the condition of the stiffness around k_E_.

Then, Matlab is used to multiply the derived angular velocity $\dot{\theta }$ and the output torque M_k_ discrete data at the sampling point, and the trapez function is used to integrate the data over a period of time. After bringing its value into equation (24), it got the energy ratio *ε* at different stiffness, as shown in Figure 12. The simulation result around the theoretical derivation value k_E_ is shown in Figure 13.

Figure 13 shows that the average moment around k = 800 N∗mm/deg is the lowest, and the maximum error between the simulated value and the theoretical value is about 1.95%.

According to the data of simulation experiments, the difference between the energy ratio in the case of nonlinear elastic joints and the energy ratio in the case of the optimal energy-saving torsion spring stiffness was first calculated. Then, calculate the ratio of the difference and the energy consumption when no elastic joint was added. It can be concluded that energy consumption of up to 52.81% can be reduced in the case of an optimal energy-saving stiffness.

### 4.3. Discussion of the Experimental Results

Chen et al. [20] use a torsional spring to participate in the movement of the quadrupedal robot joint, assisting the hydraulic drive to significantly reduce the hydraulic output during walking, thereby reducing the energy consumption. Zhou and Fu [21] propose a method based on biomechanics and bionic control strategy for the compliant quadruped robots. It mainly includes mechanical compliance element and control compliance element that can reduce the contact force both on hip and knee joints. Finally, the experiment results proved to effectively optimize joint torque and prevent damage to the robot. Ma et al. [22], inspired by small jumping animals, designed a leapfrog robot named “Grillo.” Its passive forelimbs can cushion the landing impact and can be converted into elastic potential energy and released during jumping to increase the peak power output of the motor. The above papers and the theoretical analysis of this paper and the simulation experiments indicate that adding elastic elements at certain positions of the robot joint can indeed reduce the energy consumption of the robot during walking, but may cause instability of the output torque. It may cause the decline of the stability and precision of endpoint of the robot, so it is necessary to weigh the advantages and disadvantages brought about before the introduction of the elastic joint.

## 5. Conclusions

1. This paper designs a nonlinear elastic joint and deduces it theoretically. It is proved that this design can effectively reduce the energy consumption of the legged robot when walking. The simplification of the model may bring about errors between the theoretical analysis and the simulation experiment. Even if there is an error, it can reduce about 50% of the energy consumption compared with the full-rigid joint robot without considering external friction and gravity
2. This design will reduce the order of the robot's control equation to a certain extent and increase the difficulty of control. However, for a more redundant robot, the reduction in energy consumption may increase the overall performance of the robot. This paper will be validated in later work. However, the position of the elastic joint in the overall mechanism is not flexible enough. If the joint rotation trajectory curve does not coincide with it, it may cause a reaction. That is to say, when the output torque of the joint motor accelerates the joint, the joint mechanism may come into contact with the torsion spring in advance, which causes the joint motor to drive to provide extra torque. And in the real world, there are constants of the acceleration of gravity. The upper and lower elastic joints cannot be assembled symmetrically. It is even more difficult to fit the trajectory to the elastic joints
3. The future work of this paper is to give the appropriate elastic joint assembly position and the optimal stiffness for the more general end trajectory and try to design a universal adjustable nonlinear elastic joint

## Figures and Tables

**Table 1:** Nonlinear elastic joint mechanism diagram parameters and meaning.

Parameter	Definition
*θ*	Joint ground angle
*θ*_k_	Joint and torsion spring contact angle
k_1_	The main torsion spring stiffness
k_2_	The secondary torsion spring stiffness
L	Body length
l	Half body length
M	Output torque
I	The moment of inertia of body
m	Body mass

**Table 2:** Friction parameters of rotary motion.

Parameter	Value
Mu static	0.5
Mu dynamic	0.8
Friction arm	10
Bending reaction arm	10
Pin radius	10
Static transition velocity	0.1
Max static friction deformation	0.01
Friction torque preload	0

**Table 3:** Parameter of contact force.

Parameter	Value
Stiffness	1.00E+05
Force exponent	2.2
Damping	10
Penetration depth	0.1
Friction force	Coulomb
Static coefficient	0.3
Dynamic coefficient	0.1
Static translation Vel	100
Friction translation Vel	1000

**Table 4:** The physical parameters of main torsion spring.

Spring parameter	Value
Spring stiffness k_1_ (N∗mm/deg)	400
Damping coefficient (N∗mm∗s/deg)	1
Preload (N)	0

**Table 5:** The physical parameters of secondary torsion spring.

Spring parameter	Value
Spring stiffness k_1_ (N∗mm/deg)	0.01
Damping coefficient (N∗mm∗s/deg)	0.0001
Preload (N)	0

**Table 6:** Parameters of two simulation models.

Parameter	Value
Leg length L	84.000 mm
Center of mass to axis of rotation l	47.141 mm
Equivalent body mass m	3.611E-002 kg
Equivalent moment of inertia I	107.059 kg∗mm^2^
Initial joint angle *θ*	0 deg

## Data Availability

The data that support the findings of this study are available from the corresponding author upon reasonable request.
